# Belowground Consequences of Intracontinental Range-Expanding Plants and Related Natives in Novel Environments

**DOI:** 10.3389/fmicb.2019.00505

**Published:** 2019-03-18

**Authors:** Marta Manrubia, L. Basten Snoek, Carolin Weser, G. F. Veen, Wim H. van der Putten

**Affiliations:** ^1^Department of Terrestrial Ecology, Netherlands Institute of Ecology (NIOO-KNAW), Wageningen, Netherlands; ^2^Laboratory of Nematology, Wageningen University and Research Centre, Wageningen, Netherlands; ^3^Theoretical Biology and Bioinformatics, Utrecht University, Utrecht, Netherlands

**Keywords:** ecological novelty, habitat novelty, phylogenetic distance, plant range expansion, rhizosphere community assembly

## Abstract

Introduced exotic plant species that originate from other continents are known to alter soil microbial community composition and nutrient cycling. Plant species that expand range to higher latitudes and altitudes as a consequence of current climate warming might as well affect the composition and functioning of native soil communities in their new range. However, the functional consequences of plant origin have been poorly studied in the case of plant range shifts. Here, we determined rhizosphere bacterial communities of four intracontinental range-expanding plant species in comparison with their four congeneric natives grown in soils collected from underneath those plant species in the field and in soils that are novel to them. We show that, when controlling for both species relatedness and soil characteristics, range-expanding plant species in higher latitude ecosystems will influence soil bacterial community composition and nutrient cycling in a manner similar to congeneric related native species. Our results highlight the importance to include phylogenetically controlled comparisons to disentangle the effect of origin from the effect of contrasting plant traits in the context of exotic plant species.

## Introduction

Current climate change is reshaping natural communities by enabling species range expansions to higher altitudes and latitudes (Parmesan and Yohe, 2003; Chen et al., 2011; Pauli et al., 2012). Whereas patterns for plants and animals have been relatively well explored, consequences of these range shifts for cryptic species assemblages, such as soil biota, are poorly known (Van Nuland et al., 2017). During range expansion, specific interactions between plants and their co-evolved soil organisms will become disrupted when they have different dispersal capacities (Berg et al., 2010). In the new habitat, range-expanding plant species may benefit from the absence of specialized pathogens (Engelkes et al., 2008; Van Grunsven et al., 2010b; Morriën et al., 2013; Dostálek et al., 2016). Such enemy release has been documented for exotic plant species that have been introduced from other continents (Mitchell and Power, 2003; Reinhart et al., 2010), and have been proposed to contribute to increased performance of exotics over co-occurring natives (Keane and Crawley, 2002; Blumenthal et al., 2009). However, the soil community contains not only pathogens, but also numerous other symbionts and saprophytic microbes that are involved in a variety of ecosystem processes, such as decomposition and nutrient cycling. A key question that is still not well addressed for range-expanding plant species is how multifunctional soil communities, also including saprophytic microorganisms, may respond to novel host plants with which they lack a co-evolutionary history (Van der Putten, 2012; Evans et al., 2016).

In the rhizosphere, bacterial community composition is determined by plant species and soil characteristics (Kowalchuk et al., 2002; Berg and Smalla, 2009). Saprophytic soil microbes are indirectly affected by plants through the quality and quantity of plant litter and root exudates (De Deyn et al., 2008; Eilers et al., 2010). Novel plant species that have different root exudation patterns or tissue chemistry compared to natives (Macel et al., 2014) will select a specific assemblage of belowground microorganisms (Lankau et al., 2009; Lankau, 2011). Depending on the novel plant characteristics, soil communities may shift in their composition and functions when an exotic plant species invade (Kourtev et al., 2002a; Wolfe and Klironomos, 2005; Vilà et al., 2011; Gibbons et al., 2017; Harkes et al., 2017; Mamet et al., 2017). However, many studies on belowground functional consequences of exotic invaders are based on comparing species with different traits and life history strategies. Therefore, phylogenetically controlled comparisons, which exert higher control for factors known to influence soil community composition and functioning, are important to elucidate the effects of species origin (Agrawal et al., 2005; Funk and Vitousek, 2007).

Across-species comparison has shown that the invasive potential of exotic species may result from distant relatedness to native plant species, rather than from an effect of geographical origin *per se* (Strauss et al., 2006). Therefore, identifying functional consequences of ecological novelty of exotic plant species may be more accurate when comparing exotic species with related natives. Several experimental studies have singled out effects of ecological novelty (i.e., plant geographical origin in this case) by comparing exotic plant species with congeneric natives, demonstrating that even when controlling for species relatedness exotics can differ from natives (Agrawal et al., 2005; Funk and Vitousek, 2007; Funk and Throop, 2010; Meisner et al., 2013). Here, we aim at understanding how plant species that expand their range to higher latitudes within continents will impact the composition and functioning of the soil bacterial community in the new range as a result of their ecological novelty. We determined the impact of novel range-expanding plant species on native ecosystems in comparison with congeneric natives according to a phylogenetically controlled experimental set up (Engelkes et al., 2008; Meisner et al., 2011, 2012).

In the present study, we compared rhizosphere bacterial communities of range-expanding plant species and their related natives in two different contexts; grown in soils from their own field populations and in soils where both are ecologically novel. By using these two different context, we investigated whether potential differences in rhizosphere bacterial community composition between range-expanders and native plant species are the result of selection effects by plants or a response to existing soil heterogeneity. Because differences in rhizosphere bacterial communities may depend on plant ontogeny, we repeated our assessments in a time series, so that we could control for differences that may result from plant development. Our first hypothesis was that rhizosphere community composition of range-expanding plants differs from related natives when plants grow in their “own” field soils. Our second hypothesis was that plant origin-specific differences in bacterial rhizosphere communities increase over time when plants are grown in “novel” soils, as that would reveal plant selection effects on soil communities determined by their geographical origin. We assessed functional consequences of differences in soil community composition by measuring catabolic response profiles and soil enzymatic activities. We tested our hypotheses using a controlled greenhouse experiment with four pairs of range-expanding plant species and congeneric natives. Each plant species was grown in “own” and “novel” soils. We determined bacterial community composition and community-level functioning in the rhizosphere of all plants after four, eight and twelve weeks of plant growth.

## Materials and Methods

### Plant Species Selection and Seed Origin

We used four pairs of range-expanding and congeneric native plant species and all eight species co-occur in riverine habitat of the Netherlands (Supplementary Table S1). This river-accompanying ecosystem is connected to Central Europe through the Rhine river, and to South-East Europe through the Rhine-Danube canal. In Central and South-East Europe, >800 km away from the Netherlands, the range-expanding and congeneric native plant species are all native. The plant species were selected based on the same criteria used in previous studies (Engelkes et al., 2008; Meisner et al., 2011). Briefly, we selected range-expanding plant species that are present in the Netherlands and co-occur in the same ecosystem with an abundant native plant species of the same genus. The range-expanding plant species were first recorded in the Netherlands during the second half of the 20th century with the exception of *Geranium*, which was first recorded in 19th century (Dutch flora is very well tracked by many volunteer florists) and show an increasing trend in abundance in the Netherlands over the last decades (NDFF, 2018). Because of their co-occurrence in the same riverine habitat type and their close phylogenetic (intra-genus) relationship, plant species belonging to the same pair differ in their geographical origin (i.e., range-expander vs. native), but are otherwise expected to be similar in genetic background and general ecology.

Seeds of *Rorippa* species, native *Geranium molle*, and range-expanders *Centaurea stoebe* and *Tragopogon dubius* were collected from the field in the Netherlands. Seeds of native *Centaurea jacea, Tragopogon pratensis* and the range-expander *Geranium pyrenaicum* were purchased from an external supplier that collects and propagates seeds from wild plant populations (Cruydt Hoeck, Nijeberkoop, Netherlands). All seeds were surface sterilized (3 min, 10% bleach solution) and germinated on glass beads under controlled conditions (16 h of daylight at 20°C and 8 h of darkness at 10°C). *Rorippa* seeds were not surface sterilized due to their small size and were germinated in gamma-sterilized soil (minimally 25 KGray, Syngenta BV, Ede, Netherlands).

### Soil Collection

During August–October 2015, we collected soils from five independent plant populations of each plant species in order to act as soil inocula in our experiment (Supplementary Table S1). All soils were sampled from field populations in the new range. Therefore, initial differences in soil community composition could not have been the result from environmental differences between the original and new range. A total of 40 inoculum soils were collected and kept separate as five experimental replicates throughout the experiment. Even though the plant species of interest occurred in mixed plant communities, soils were collected from underneath individuals of the species of interest in each of the five populations. Soil samples were collected from locations that were at least 60 m apart from each other (Supplementary Table S1). Field soils were sieved using a 4 mm mesh size to remove coarse elements and stored at 4°C in the dark until the experiment started. A subsample of each soil was stored at –80°C for further molecular analyses of the soil microbial community. A second subsample was oven dried at 40°C for 5 days in order to determine moisture content and soil C:N ratio using an elemental analyzer (Flash EA 1112, Thermo Fisher Scientific Inc., Waltham, MA, United States). Soil available phosphate (P-Olsen) was extracted in a 0.5 M NaHCO_3_ solution and quantified using an autoanalyzer (QuAAtro Autoanalyzer, SEAL Analytical Ltd., Southampton, United Kingdom). Finally, we extracted available N (nitrate and ammonia) from field moist soils by shaking a 10 g dry weight equivalent in a 50 ml of 1 M KCl solution for 2 h. We measured soil pH in the KCl extracts and determined the concentration of mineral nitrogen (NH_4_^+^ and NO_3_^-^–NO_2_^-^) using an autoanalyzer (QuAAtro Autoanalyzer, SEAL Analytical Ltd., Southampton, United Kingdom).

In our experiment, we inoculated a sterilized background soil with living (non-sterilized) field soil. This method is commonly used and allows studying plant responses to soil biota while controlling for potential differences in abiotic properties of the soils (Engelkes et al., 2008). Background soil was collected from a riparian area near Beneden-Leeuwen, Netherlands (N51°53.952, E05°33.670). Background soil was sieved using a 1 cm mesh size, homogenized and gamma-sterilized (minimally 25 KGray, Syngenta BV, Ede, Netherlands).

### Experimental Setup

**FIGURE 1:**
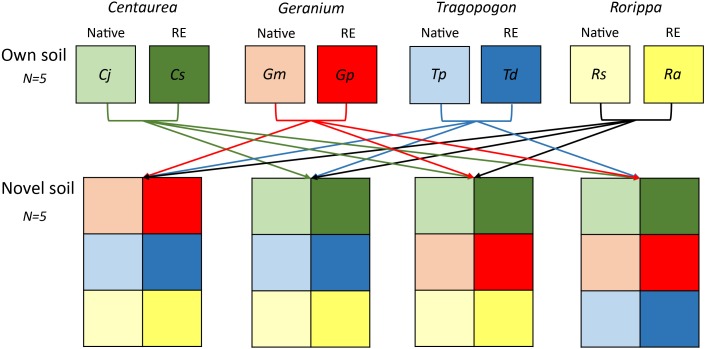
Scheme of soil inocula treatments used in the experiment. Each plant species was grown in sterile soils inoculated with live soil originating from underneath plant individuals in the field (own soils). Furthermore, each species of each congeneric pair was grown in sterile soils inoculated with a mixture of live soils origination from underneath the non-congeneric species in the field (novel soils). A total of 5 independent soil live inocula replicates were collected from the field and kept separate throughout the experiment.

We inoculated the sterilized background soil with 10% of live field soil (dry weight basis). We grew each plant species in soils inoculated with two different types of soil inocula: an inoculum from field sites where the plant species was present in the field (“own” soils), and an inoculum that is novel to the plant species (“novel” soils) (Figure 1). Novel soils for each congeneric plant pair were created by mixing the soils of all non-congeneric species using equal amounts on a dry weight basis. For both “own” and “novel” soils there were five independent replicates (Figure 1). Therefore, the “novel” soil mixes also originated from habitats within the riverine ecosystem where plant species could occur, but had not been previously conditioned by the plant species grown in the experiment. The novel soil of each replicate was split into two halves, one for growing the range expander and the other for growing the congeneric native.

Pots of 1.1-L were filled with the equivalent of 850 g of dry weight of soil. We adjusted soil moisture to 60% of the soil water holding capacity and kept it constant during the experiment by watering two times per week to re-set weight. Pots with soil only were pre-incubated in the greenhouse for 4 days in order to adjust to the water content and allow the inoculated soil microbial communities to establish in the sterilized soil. Afterwards, one seedling of each plant species was planted in the pots. During the first week, we replaced the seedlings that failed to establish. Greenhouse conditions represented an average growth season in the new range and were controlled to 16 h day length with day temperature of 21°C, night temperature of 16°C and average air humidity of 60%. Artificial light was supplied when required [High pressure Sodium (Son-T, 600 W Philips GP)].

We destructively sampled rhizosphere soil at 3 different time points during plant development and measured plant biomass after 4, 8, and 12 weeks since the start of the experiment. Pots were organized in a randomized block design in the greenhouse with 5 replicate blocks. In total, 240 pots were set up (8 plant species × 2 soil treatments (“own” and “novel”) × 3 time points × 5 replicates).

### Soil and Plant Biomass Sampling

At each sampling time, we destructively harvested 80 pots (8 plant species × 2 soil treatments (“own” and “novel”) × 5 replicates). First, we removed the whole plant and soil from the pot. Then, the top soil in the pots and the soil attached loosely to plant roots was separated and discarded. Finally, roots were shaken vigorously and the soil that detached last from the roots was considered the “rhizosphere soil.” We filled an Eppendorf tube with that rhizosphere soil and stored it at –80°C for further molecular analyses of the bacterial community composition. We used the remaining rhizosphere soil to analyze soil community functioning. Plant roots and shoots were separated, roots were washed and above and belowground plant biomass was measured after oven drying to constant weight at 70°C for 48 h (data not shown).

### DNA Extraction and Community-Level Sequencing Analyses

We extracted DNA from all soil samples and subsequently amplified the 16S rRNA gene to determine bacterial community composition. Eppendorf tubes containing rhizosphere soil were freeze-dried prior to DNA extraction (FreeZone 12, Labconco, Kansas City, MO, United States). DNA was extracted from 0.25 g of soil using PowerSoil DNA Isolation Kit (Mo Bio Laboratories, Carlsbad, CA, United States) following the manufacturer’s instructions. We then amplified DNA using duplicate PCR reactions with bar-coded primers. Bacterial community composition was determined by targeting 16S rRNA gene using 515F/806R primers (Caporaso et al., 2012). PCR products were purified using the Agencourt AMPure XP magnetic bead system (Beckman Coulter Life Sciences, Indianapolis, IN, United States) with a volume of PCR product to beads of 1 to 0.7. Purified PCR products were analyzed in a Fragment Analyzer using a Standard Sensitivity NGS Fragment Analysis kit (1–6000 bp) and following manufacturer’s instructions (Advanced Analytical Technologies GmbH, Heidelberg, Germany). Finally, bacterial PCR amplicons were sequenced using Illumina MiSeq platform.

The 16S rRNA amplicon reads, MiSeq paired-end reads, were merged when reads had a minimum overlap of 150 bp and at least a PHRED score of 25 using the RDP extension of PANDASeq (Masella et al., 2012) named Assembler (Cole et al., 2014). Primer sequences were removed using Flexbar version 2.5 (Dodt et al., 2012). Sequences were clustered to OTU with VSEARCH version 1.0.10 (Rognes et al., 2016), using the UPARSE strategy by dereplication, sorting by abundance with removing singletons and clustering using the UCLUST smallmem algorithm (Edgar, 2010). Chimeric sequences were detected using the UCHIME algorithm. All reads were mapped to OTUs and an OTU Table was created and converted to BIOM-Format 1.3.1 (McDonald et al., 2012). Taxonomic information for each OTU obtained using the RDP Classifier version 2.10 (Cole et al., 2014). All steps where implemented in a workflow made with Snakemake (Köster and Rahmann, 2012). Samples with a total sequence number lower than 1000 reads and singleton OTUs (e.g., OTU which is only found once in one sample) were removed from further analyses. The sequencing analyses of the 16S rRNA region of all soils yielded an average of 2.761 OTUs per sample (±944 SD), with a total of 5.452.733 reads. OTUs belonged to 25 different phyla (including bacteria and archaea), 82 classes, 136 orders, 274 families and 630 genera.

### Catabolic Response Profile of the Soil Community

We used a catabolic response profile method as described in Fierer et al. (2012) in order to assess how soil communities differ in their ability to mineralize different organic carbon compounds. For each pot, we measured the CO_2_ production response of the soil communities after the addition of 8 organic substrates of varying complexity (i.e., glucose, sucrose, glycine, oxalic acid, citric acid, yeast, lignin and cellulose). Organic carbon solutions were made before the soil sampling started and adjusted to a pH of 6.0. These analyses were carried out immediately after sampling of rhizosphere soil. Briefly, the equivalent of 4 g of dry soil was weighed into 9 different 50 ml centrifuge tubes. Then, each tube received 8 ml of one of the organic carbon substrate solutions. Additionally, one of the tubes received water as a control. Tubes containing soils and substrates were incubated for 1 h uncapped in a horizontal shaker (20°C). Centrifuge tubes were then closed tightly with a modified lid equipped with a rubber septum and a rubber O-ring in order to ensure air tightness. We then flushed the headspace air in the tubes with CO_2_-free air for 2 min at 1 bar (Westfalen Gassen Nederland BV, Deventer, Netherlands). We incubated the tubes at constant temperature of 20°C in the dark using a climate-controlled chamber (Economic Lux chamber, Snijders Labs, Tilburg, Netherlands).

After incubation, we collected 6.2 ml of headspace air from each tube using a syringe and stored it into pre-evacuated 5.9 ml Exetainer vial (Labco Ltd., Buckinghamshire, United Kingdom). Samples were collected after 4 h of incubation for the water control and the labile substrates (glucose, sucrose, glycine, oxalic acid, citric acid and yeast) and after 24 h for more recalcitrant substrates (lignin, cellulose). The concentration of CO_2_ in the gas vials (over pressure of 1 bar) was measured by injecting 250 μl of each sample in a Trace Ultra GC gas chromatograph equipped with a flame ionization detector with methanizer (mFID) (Interscience BV, Breda, Netherlands) and a TriplusRSH auto-sampler (Interscience BV, Breda, Netherlands), and a Rt-QBOND (30 m, 0.32 mm ID) capillary column (Restek, Bellefonte, PA, United States). We used helium 5.0 as a carrier gas, a sample split ratio of 1:20 and set oven temperature at 50°C with a flow of 5 ml. We used a calibration curve of known concentrations of CO_2_ ranging from 0 to 4600 ppm of CO_2_ prepared out of a reference gas (2.38% CO2 in synthetic air, Westfalen AG, Munster, Germany) to determine the amount of CO_2_ in our samples. Chromeleon 7.2 Data System Software (Thermo Fisher Scientific, Waltham, MA, United States) was used to automatize the measurements and process data. Respiration profiles were determined for each sample by calculating the relative respiration response from each of the incubations (in the control and the 8 different substrate additions) with respect to the total amount of respiration measured for that sample.

### Extracellular Hydrolytic Enzyme Activity

Remaining rhizosphere soil was kept at –20°C for further analyses of extracellular enzyme activity in the soil. We measured soil enzyme activity using high-throughput fluorometric measurements, where a gain of fluorescence over the incubation time represents the amount of enzymatic activity (Baldrian, 2009). We determined the potential activity of 3 enzymes in soils involved in different pathways of carbon and nutrient cycling: glucosidase, phosphatase and aminopeptidase. Enzyme activity was measured in the 80 soil samples of the last time point (12 weeks). Briefly, 1 g of fresh soil was weighed into a clean glass jar before adding 50 ml of sodium acetate buffer (2.5 mM, pH 5.5). Vials were then capped tightly and shaken in a horizontal shaker for 10 min at 330 rpm in order to obtain the soil homogenate. Fluorogenic substrates 4-methylumbellyferyl-β-D-glucopyranoside (MUFG), 4-methylumbellyferyl-phosphate (MUFP) and L-alanine-7-amido-4-methylcoumarin (AMCA) were purchased (Sigma-Aldrich Chemie NV, Zwijndrecht, Netherlands). We dissolved all substrates in DMSO at concentration of 2.5 mM for AMCA and 2.75 mM for MUFG and MUFP. A 40ul of substrate solution was mixed with 250 μl of soil homogenate in each well of a black 96-well plate. Three technical replicates were included per soil sample and enzyme activity. We calibrated concentrations of enzyme activity product by a dilution curve made from a stable form of the fluorogenic compounds (1.0 mM methylumbellyferol (MUF) and 1.0 mM 7-aminomethyl-4-coumarin (AMC) (Sigma-Aldrich Chemie NV, Zwijndrecht, Netherlands). Fluorescence was measured at time 0 h and after 2 h of incubation at 40°C. We used a 96-well plate reader with an excitation and emission wavelengths of 360 and 460 nm, respectively (Synergy HT, BioTek Instruments, Winooski, VT, United States). We compared the measured fluorescence in our samples, after subtraction of the blank, with standard curves of MUF and AMC to calculate the amount of enzymatic product formed over the incubation time. A unit of enzyme activity is defined as the amount of enzyme reaction product (μmol) per gram of dry soil and hour.

### Statistical Analyses

Abiotic properties of field inocula soils were analyzed with 2-way ANOVA in R (R Core Team, 2017). We tested the effect of plant genera and plant origin on each of the soil abiotic parameters. We considered plant genus as a fixed factor and not random since we selected the genera available after accounting for our selection criteria (Engelkes et al., 2008; Meisner et al., 2011). We tested the effect of plant origin within each plant genus for each of the soil parameters using *post hoc* comparisons of least square means with Tukey adjustment. Data was transformed prior analyses to meet assumptions of normality using Box-Cox power transformation for linear models in R (R Core Team, 2017).

Canoco 5 software was used to conduct multivariate statistics on bacterial community composition of field soils used as inoculum, and on compositional and functional (CRP) data of rhizosphere bacterial communities (Ter Braak and Šmilauer, 2012). Relative abundances of bacterial OTUs in soil communities and on soil respiration responses were log transformed prior the analyses. We performed Principal Coordinate Analyses (PCoA) of the dissimilarity matrix based on Bray–Curtis distances to visualize differences in bacterial community composition and soil functioning between our treatments. For the inoculum soils, soil abiotic properties measured (pH, C:N ratio, nitrate, ammonia and plant-available phosphate) were projected as (supplementary variables) in the PCoA ordination. Furthermore, we statistically tested the effect of the soil parameters measured and plant species on bacterial community composition of the inoculum soils using PERMANOVA (9999 permutations) (Oksanen et al., 2018). For the experimental soils, we tested the effect of plant genera, plant origin, soil and time point on bacterial community composition and community functioning using PERMANOVA (9999 permutations) (Oksanen et al., 2018). As explained earlier in the Methods section, during the experiment, each plant pair formed by a native and range-expanding plant species was grown in novel soils created by mixing the soils from the replicate sites of the non-congeneric plant species. Thereby, the novel soils were different from one plant pair to another and, for this reason data analyses were performed for each plant pair separately. Permutation tests were performed within each plant pair and we tested the effect of individual and interaction effects of plant origin, soil inocula and time of soil conditioning by the plants. For the same reason, we also conducted the analyses in “novel” and “own” soils per plant pair separately. The analyses within “novel” and “own” soils allowed us to zoom in on the plant-driven variation in rhizosphere communities and their functions in the case of novel soils, and examine differences in more detail in the case of own soils. To statistically test the significance of plant origin, soil inocula and time of harvest effects on community composition and functioning, we performed PERMANOVA analyses (9999 permutations) using the “adonis” function in the “vegan” package in R (Oksanen et al., 2018). We performed pairwise comparisons using the “pairwiseAdonis” function (Martinez Arbizu, 2017). In all cases, block was included as a covariate in the analyses. We also performed multivariate dispersion analyses (999 permutations) to test for homogeneity of dispersion between the different plant origin, soil and time point groups and, thereby, validate the PERMANOVA tests (Anderson, 2006). Homogeneity of dispersion was measured using the “betadisper” function in the “vegan” package (Oksanen et al., 2018).

Bacterial OTU richness and Shannon’s diversity indices of bacterial communities (H’) and evenness were computed for each sample using Canoco 5 (Ter Braak and Šmilauer, 2012). These community parameters were determined in inocula soils and analyzed in the same way as the abiotic soil properties described above. For the experimental soils, we analyzed community parameters with linear mixed models using “lmerTest” package (Kuznetsova et al., 2017). We modeled community parameters for each of the three time points separately with plant genus, plant origin and soil as fixed effect factors and block as random factor. All analyses were conducted in R (R Core Team, 2017). OTU richness was log transformed prior to analyses. Diversity and evenness data were transformed prior to analyses to meet assumptions of normality using Box-Cox transformation for linear models in R.

Additionally, the effects of plant origin and soil inocula on soil enzyme activity were tested using linear mixed models with plant genus, plant origin (native or range expander) and soil inocula (“own” or “novel”) as fixed effect factors, and block as a random factor. Enzyme activity rates were log transformed prior to analyses to meet assumptions of normality. All analyses were conducted in R (R Core Team, 2017).

## Results

### Bacterial Community in Field Soils (Inocula Soils)

The variation of bacterial community composition represented by the first two axes of the PCoA was 39% of the total variation (Supplementary Figure S1). Soil abiotics and plant species identity explained 40 and 21% of the variation in soil bacterial communities, respectively, as tested with PERMANOVA (Supplementary Table S2). Among soil abiotic properties measured, soil pH explained the largest amount of variation in soil bacterial communities (Supplementary Table S2). Bacterial communities in field soils of native and range-expanding *Geranium* species were more similar to each other than for the other plant pairs. However, soil bacterial communities associated with the natives *C. jacea* and *T. pratensis* were more similar to each other than to their related range-expanders *C. stoebe* and *T. dubius* (Supplementary Figure S1). Soil bacterial communities of *Rorippa* species were most different from the rest and were associated to soils with higher soil pH and C:N ratio compared to the other plant species (Supplementary Figures S1, S2 and Supplementary Table S3). Overall, bacterial community richness, diversity and evenness was affected by plant genus, yet no significant effect was found for plant origin (Supplementary Table S3 and Supplementary Figure S2). Soil nitrate availability was significantly higher in soils of native plant species than in soils of range expanders. In the case of ammonia, soils of native species had lower ammonia than soils of range-expanders, with the exception of the *Tragopogon* species (Supplementary Table S3 and Supplementary Figure S2).

### Bacterial Community Composition in the Rhizosphere of the Experimental Plants

**FIGURE 2:**
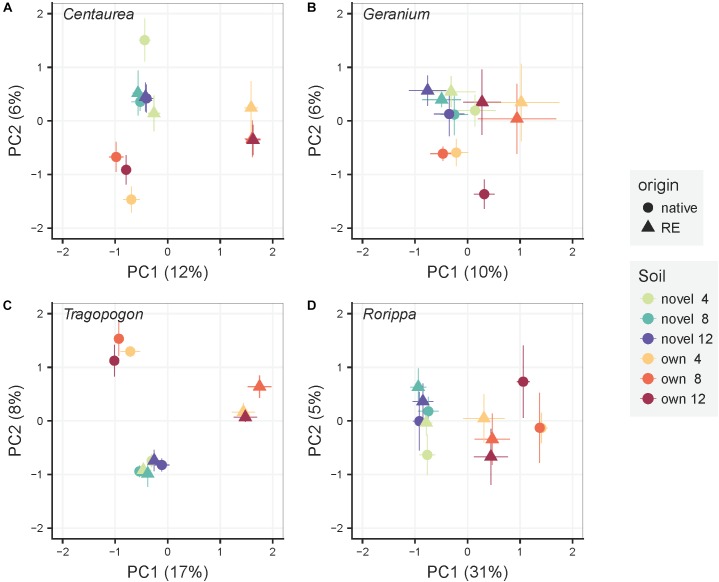
Principal Coordinate Analyses of the rhizosphere bacterial community composition for each pair of a range-expander and its congeneric native plant species (**A**: *Centaurea*, **B**: *Geranium*, **C**: *Tragopogon*, **D**: *Rorippa*) grown in soils from their own field locations (own) and soils that are novel to both of them (novel). The symbols are means ± SE (*N* = 5). Within each pair, circles represent the native plant species and triangles the range-expander. Colors indicate soil treatment (“own” and “novel”) and time of harvest (4, 8, and 12 weeks) as noted in the legend.

The variation of bacterial community composition represented by the first two axes of the PCoA accounted for 18, 16, 25, and 36% of the total variation in *Centaurea, Geranium, Tragopogon* and *Rorippa* species, respectively (Figure 2). In the overall PERMANOVA test, the effect of plant origin on rhizosphere bacterial community was interacting with plant genera and soil (Supplementary Table S4). Furthermore, there was a main effect of time of sampling. Bacterial communities after 4 weeks of plant growth significantly differ from those samples at week eight and twelve of the experiment (*p* = 0.002 and *p* = 0.001, respectively), while there were no differences between the latter time points as indicated by pairwise testing. When considering plant pairs separately, soil inocula (“novel” or “own”) was the most important factor explaining variation in bacterial community composition in all plant pairs with the exception of the *Tragopogon* pair, where soil inocula and plant origin explained the same amount of variation (Table 1). The interaction of plant origin and soil inoculum explained bacterial community in the rhizosphere in all plant pairs except in *Geranium* (Table 1). Overall, bacterial communities were separated between native and range expanders when they were grown in their “own” field soils, but did not differ when grown in “novel” soils. Multivariate dispersion analyses indicated that dispersion within groups was homogeneous between range-expanders and natives and between time points for each of the four plant pairs. However, “own” soils had significantly higher dispersion than “novel” soils in *Geranium, Tragopogon* and *Rorippa* plant pairs (*F* = 8.8, *p* = 0.004, *F* = 58.2, *p* = 0.001 and *F* = 28.8, *p* = 0.001, respectively). This indicated that community composition was most similar between samples of “novel” soils, while there was most variation between samples in “own” soils. As a result of the soil mixing scheme (Figure 1), own soils for each plant pair consisted of ten different soils originating from individual locations in the field, causing a higher multivariate variation, while novel soils consisted of five different soil mixes from locations of the non-congeneric species in the field.

**Table 1:** PERMANOVA tests on Bray–Curtis dissimilarity matrix (9999 permutations).

		*Centaurea*		*Geranium*		*Tragopogon*		*Rorippa*	
					
	Factor	*R*^2^	Signif.	*R*^2^	Signif.	*R*^2^	Signif.	*R*^2^	Signif.
Bacterial community	Plant origin (P)	**0.057**	^∗∗∗^	**0.036**	^∗∗^	**0.082**	^∗∗∗^	**0.030**	^∗^
	Soil inocula (S)	**0.067**	^∗∗∗^	**0.049**	^∗∗∗^	**0.082**	^∗∗∗^	**0.199**	^∗∗∗^
	Time (T)	**0.050**	^∗∗^	**0.054**	^∗∗^	**0.047**	^∗^	0.040	ns
	P × S	**0.055**	^∗∗∗^	0.023	ns	**0.081**	^∗∗∗^	**0.026**	^∗^
	P × T	0.020	ns	0.024	ns	0.022	ns	0.018	ns
	S × T	0.022	ns	0.021	ns	0.021	ns	0.023	ns
	P × S × T	0.019	ns	0.019	ns	0.023	ns	0.016	ns

Community functioning	Plant origin (P)	0.003	ns	0.001	ns	0.001	ns	0.000	ns
	Soil inocula (S)	0.031	ns	0.028	ns	0.028	ns	0.028	ns
	Time (T)	0.009	ns	0.005	ns	0.009	ns	0.010	ns
	P × S	0.002	ns	0.001	ns	0.000	ns	0.000	ns
	P × T	0.001	ns	0.000	ns	0.000	ns	0.001	ns
	S × T	0.000	ns	–0.001	ns	–0.001	ns	0.000	ns
	P × S × T	0.001	ns	0.000	ns	0.000	ns	0.001	ns


*Significance levels: ns *p* > 0.05; ^∗^*p* < 0.05; ^∗∗^*p* < 0.01; ^∗∗∗^*p* < 0.001; ^∗∗∗∗^*p* < 0.0001. Values in bold indicate *p* < 0.05.*

#### Bacterial Communities Within “Novel” and “Own” Soils

To test the effect of plant origin more accurately we performed the same analyses within each soil inocula treatment, which allows to disentangle the effect of plant origin from the effect of pre-existing differences in soil bacterial communities. We then observed that, in “novel” soils, plant origin no longer explained variation in bacterial community composition (Supplementary Figure S3 and Supplementary Table S5). Instead, time of harvest appeared to explain around 10% of the total variation in *Geranium* and *Tragopogon* plant pairs (Supplementary Table S5). In “own” soils, i.e., originating from field populations of each species, plant origin significantly explained bacterial community composition at the end of the growth experiment (Supplementary Figure S4 and Supplementary Table S5). In both “novel” and “own” soils, the variation in community composition of the samples belonging to range-expanders and natives and to the different time points showed homogeneous multivariate dispersion, indicating that the variation in community composition was equal between the groups. There was only the exception of the *Geranium* plant pair grown in “own” soil, where soils of range-expanders had higher variation in community composition than soils of natives (*F* = 5.8, *p* = 0.02).

#### Bacterial Community Richness, Diversity and Evenness

Soil treatments (“own” and “novel”) significantly differed in their bacterial richness and diversity at all sampling times (Supplementary Table S6; Fixed factors, Soil). Novel soils, which were mixes of all soil samples collected from field sites with non-congeneric plant species, had significantly higher richness and diversity of bacterial OTUs than own soils, which originated from individual locations where the tested plant species were present in the field. Bacterial communities in own soils of *Rorippa* species were most different from the other plant pairs (Supplementary Figure S2 and Supplementary Table S6) and also from their novel soil, which is represented by a significant soil and plant genera interaction. Overall, richness, diversity and evenness of rhizosphere bacterial communities were not significantly affected by plant origin itself (Supplementary Table S6).

### Community-Level Functional Analyses

#### Catabolic Response Profile

**FIGURE 3:**
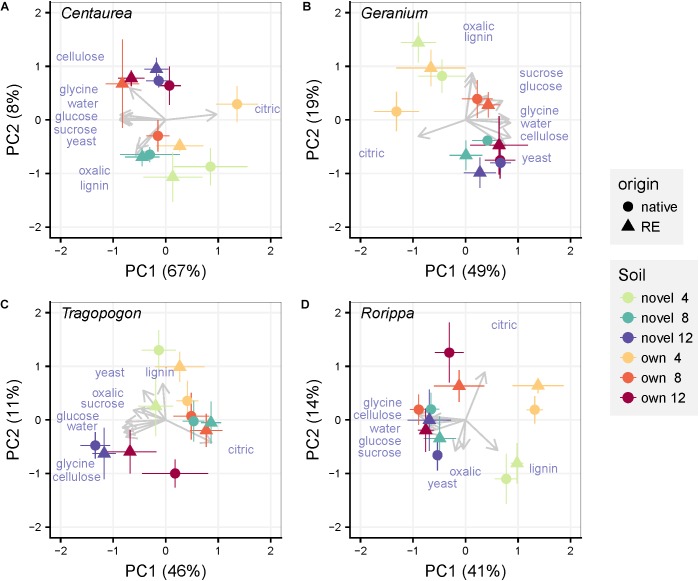
Principal Coordinate Analyses of the catabolic response profiles for each pair of a range-expander and its congeneric native plant species (**A**: *Centaurea*, **B**: *Geranium*, **C**: *Tragopogon*, **D**: *Rorippa*) grown in soils from their own field locations (own) and soils that are novel to both of them (novel). The symbols are means ± SE (*N* = 5). Within each pair, circles represent the native plant species and triangles the range-expander. Arrows representing each substrate are displayed over the ordination plot as supplementary variables. Colors indicate soil (“own” and “novel”) and time of harvest (4, 8, and 12 weeks) as noted in the legend.

We used PCoA ordination to assess differences in the functional responses of soil communities to the various added organic substrates (catabolic response profile). The first and second axis represented 75, 68, 57, and 55% of the variation in catabolic response profiles of *Centaurea, Geranium, Tragopogon* and *Rorippa* soil communities, respectively (Figure 3). In the overall PERMANOVA test, there was only a significant effect of time point on explaining variation in soil community functioning (Supplementary Table S4). However, this effect of time point was weak and pairwise *post hoc* testing resulted non-significant. Even though time seems to drive dissimilarity in community functioning in the ordination PCoA plots of each plant pair (Figure 3), none of the experimental treatments (plant origin, soil inocula, time of harvest) explained differences in community level functioning (Table 1). Similarly, when functioning of the “novel” or “own” soils were examined separately, neither plant origin nor time of harvest explained the variation in community level functioning (Supplementary Figures S5, S6 and Supplementary Table S4). Overall, compositional differences in bacterial communities were not consistently linked to shifts in catabolic response profiles in our experiment. Multivariate dispersion analyses for community functioning showed that variation among functional profiles was not significantly different between groups of samples with the same plant origin, soil inoculum type and across time points.

#### Extracellular Enzyme Activity

To study soil functions related to nutrient cycling, the activity of three extracellular enzymes was measured in the rhizosphere soil collected after 12 weeks of plant growth (Table 2). There were no main effects of plant origin, indicating that both range-expanders and related native species were associated with the same levels of enzyme activity in their rhizosphere soil. Plant genus marginally affected glucosidase enzyme activity in the soil (*F*_3,60_ = 2.91, *p* = 0.04); however, *post hoc* testing using Tukey HSD did not yield significant differences between any specific group. Soil treatment (“own” and “novel”) significantly affected phosphatase activity (*F*_1,60_ = 11.00, *p* = 0.001), with higher levels of phosphatase activity in “own” than in “novel” soils.

**Table 2:** Hydrolytic enzyme activity in the rhizosphere soil after 12 weeks of plant growth. Values are means ± SE (*N* = 5).

Plant genus	Plant species	Plant origin	Soil inocula	Glucosidase activity (μmol * kg^-1^ * h^-1^)	Phosphatase activity (μmol * kg^-1^ * h^-1^)	Aminopeptidase activity (μmol * kg^-1^ * h^-1^)
*Centaurea*	*C. jacea*	Native	own	10.18 ± 1.89	10.81 ± 0.34	0.77 ± 0.16
			novel	8.78 ± 2.25	9.73 ± 1.53	0.68 ± 0.12
	*C. stoebe*	Range-expander	own	10.43 ± 1.41	10.60 ± 1.49	1.27 ± 0.19
			novel	9.98 ± 1.67	8.77 ± 1.22	0.58 ± 0.06
*Geranium*	*G. molle*	Native	own	15.73 ± 3.16	21.14 ± 5.83	1.32 ± 0.25
			novel	12.50 ± 0.68	15.90 ± 9.98	1.47 ± 0.25
	*G. pyrenaicum*	Range-expander	own	18.32 ± 4.36	22.79 ± 5.68	1.38 ± 0.13
			novel	10.50 ± 2.39	9.18 ± 2.15	1.56 ± 0.46
*Tragopogon*	*T. pratensis*	Native	own	13.12 ± 1.24	13.36 ± 2.02	0.91 ± 0.25
			novel	14.45 ± 1.79	12.63 ± 0.63	1.59 ± 0.57
	*T. dubius*	Range-expander	own	10.51 ± 1.24	17.23 ± 2.52	0.95 ± 0.09
			novel	11.66 ± 1.83	12.23 ± 1.68	0.90 ± 0.17
*Rorippa*	*R. sylvestris*	Native	own	11.54 ± 1.48	13.59 ± 1.71	1.14 ± 0.53
			novel	9.89 ± 2.25	9.58 ± 1.92	0.96 ± 0.21
	*R. austriaca*	Range-expander	own	12.82 ± 2.02	17.35 ± 3.80	1.41 ± 0.43
			novel	13.75 ± 1.61	11.40 ± 3.55	3.07 ± 1.54

Fixed factors		Plant genus	*F*-value	2.915	2.664	2.566
			*p*-value	**0.041**	0.056	ns
		Plant origin	*F*-value	0.035	0.068	0.524
			*p*-value	ns	ns	ns
		Soil inocula	*F*-value	2.108	11.008	0.227
			*p*-value	ns	**0.001**	ns


*Effects of plant genus, plant origin and soil inocula (“own” and “novel”) on enzyme activity were analyzed using ANOVA with block as random factor in the mixed linear models. Significance levels of 2-way and 3-way factor interactions resulted all non-significant and are not included in this table. Values in bold indicate *p* < 0.05*.

## Discussion

It is generally assumed that exotic plant species may alter composition and functioning of soil microbial communities (Ehrenfeld et al., 2001; Allison et al., 2006; Ehrenfeld, 2010). However, in most case studies where exotics are compared with natives, the exotics that replace the natives not only differ in origin, but also in traits or life histories, whereas pre-invasion site conditions cannot easily be controlled for (Kourtev et al., 2002a; Wolfe and Klironomos, 2005; Vilà et al., 2011). Here, we compare how intra-continental range expanding plant species and congeneric natives influence bacterial community composition and functioning in their rhizosphere, while minimizing genetic differences between range expanders and natives, and controlling for ecological novelty. We paired plant species that expand range most likely as a result of climate warming with species from the same genus that are native in the expansion range (Engelkes et al., 2008; van Grunsven et al., 2010a; Meisner et al., 2011; Morriën et al., 2013). All plant species were grown in soils collected from established field populations, as well as in soils from sites where neither the range expander nor the native currently occurred. In support of our first hypothesis, rhizosphere bacterial communities differ between range-expanding and native plant species when plants are grown in soils collected from their own field populations. Interestingly, when both species were grown in novel soils, there were no compositional differences in rhizosphere bacterial communities. Therefore, and opposite to our second hypothesis, we argue that in the present comparison plant origin *per se* has little effect on rhizosphere bacterial community composition.

Many field studies on plant invasions and soil communities have shown that exotic plant species have distinct soil communities compared to native plants growing in adjacent areas (Kourtev et al., 2002a,b; Scharfy et al., 2009; Collins et al., 2016; Stefanowicz et al., 2016; Gibbons et al., 2017). Consistent with these results, in our experiment we also observe that the composition of the rhizosphere bacterial community differed by plant origin in all four plant pairs when plants were grown in their own field soils (Figure 2 and Supplementary Figure S4). These results suggest that plant origin indeed influences bacterial community composition even in a phylogenetically constrained comparison between range expanders and natives. However, in the present study, as well as in most field studies it is difficult to exclude the possibility that invaded sites were already different from adjacent sites prior arrival of the exotic species. If that is the case, the bacterial assembly in the rhizosphere may simply reflect differences of initial bulk soil communities (de Ridder-Duine et al., 2005), or a combination of site and origin differences and, overall, the capacity to disentangle the effect of ecological novelty from other co-varying effects is limited. We tried to rule out such confounding factors as much as possible by growing range-expanding and native plant species also in the same “novel” soil.

In contrast to our second hypothesis, the geographical origin of the plant species (range-expanding or native) did not affect rhizosphere bacterial community composition differently when both range-expanding and congeneric native plant species were grown in the same “novel” soils (Figure 2 and Supplementary Figure S3). Our results diverge from previous controlled experiments, which concluded that plant origin may play a role in influencing soil community composition in both intercontinental exotic plant invasion (Kourtev et al., 2003) and plant range-expansion (Morriën et al., 2013). In contrast with many intercontinental exotic plant invasion studies, we used phylogenetically controlled comparisons to assess the effect of plant species origin, while intending to minimize their ecological differences. Consequently, plant species in each plant pair were not expected to differ strongly in e.g., life history and plant functional type, which have been suggested as important biotic predictors of soil community and soil functional shifts (Scharfy et al., 2011; de Vries et al., 2012; Legay et al., 2014; Lee et al., 2017).

The relatively short running time of our experiment (3 months) will not have allowed divergence of the bacterial communities between plants from different origins. However, the running time of our experiment is not shorter than that of most plant-soil feedback experiments where plants produced different soil microbial community structure and composition (Morriën et al., 2013; Heinen et al., 2017). In addition, *Geranium* and *Rorippa* species started to senesce within the 12 weeks of experiment, so that the length of the growth period was natural. Furthermore, the value of this 12 weeks experiment is that it reveals the microbiome response to plant identity (structural and chemical plant traits) without much influence of evolutionary dynamics between plants and soil microbial communities (Lankau, 2012). Furthermore, our main interest was to assess shifts in saprophytic microbes in the rhizosphere community and thereby, we analyzed the composition of the whole bacterial community rather than looking at the abundance of specific microbial groups, such as potential plant pathogens (Morriën et al., 2013). Seed surface sterilization might have also influenced bacterial community composition in the rhizosphere compared to when using unsterilized seeds. However, it allowed to disentangle the selection effects of plants on soil bacteria in the rhizosphere from the differences that may be caused by the pre-existing community in the seed surface. Furthermore, in a recent study it has been shown that seed-borne endophytic oomycetes of these range-expanding plant species and congeneric natives could not be found in the rhizosphere (Geisen et al., 2017) suggesting a limited effect of endophytic microbial community to the composition of the rhizosphere.

In spite of the substantial differences in bacterial community composition in the rhizosphere of plants growing in soil collected from field populations (Figure 2), we observed limited differences in community functioning (Figure 3 and Table 2). Previous experiments manipulating the composition of microbial communities have shown high levels of functional redundancy in microbial communities (Franklin and Mills, 2006; Wertz et al., 2006). Plant-induced changes in microbial-mediated soil processes may be the result of comparing phylogenetically distant plant species (Ehrenfeld et al., 2001) or major plant community shifts (Carney and Matson, 2005). They may also be derived from studies focusing on longer time scales (Collins et al., 2016) or on specific soil processes such as nitrogen cycling (Hawkes et al., 2005). Nevertheless, in spite of the possibility that plant selection effects may have influenced soil fungal communities (Dassen et al., 2017; Hannula et al., 2017), it is obvious that those changes, if occurring in our study, have not influenced soil microbial functioning either. Although the results of our experiment are consistent across the four plant pairs examined, we stress that future studies should test for consistency of our results by repeating such manipulative experiments under similar and contrasting environmental conditions.

We conclude that intracontinental plant range expansions may lead to populations of novel plant species that have different bacterial communities than congeneric natives, but that this is not necessarily due to their different geographical origin. When range expanding and native plant species from the same genus pair were grown for 3 months in novel soils, bacterial rhizosphere communities of the range expander and the congeneric native were indistinguishable. Interestingly, the differences in bacterial community composition when plants were grown in their own soils did not result in altered ecosystem processes as is demonstrated by the respiration of different organic substrates. Therefore, our results demonstrate that plant origin *per se* does not necessarily have a major impact on bacterial community composition and soil microbial functioning when keeping all other aspects the same. This does not exclude the possibility that range expanders may influence community composition and ecosystem functioning when they are exposed to the soils for longer time periods, or in other ways, such as by responding differently to extreme weather events (Meisner et al., 2013), natural enemies (Engelkes et al., 2008; Van Grunsven et al., 2010b; Dostálek et al., 2016), and other conditions that may typify their novel environments.

## Data Availability

All sequence data has been uploaded to the European Nucleotide Archive (ENA) under the accession number PRJEB26590.

## Author Contributions

MM, GFV, and WP designed the study and wrote the manuscript with input from all authors. MM, GFV, and CW performed the experiments and collected the data. MM and LBS analyzed the data with input from GFV and WP.

## Conflict of Interest Statement

The authors declare that the research was conducted in the absence of any commercial or financial relationships that could be construed as a potential conflict of interest.
