# Supplementary material for: Belowground Consequences of Intracontinental Range-Expanding Plants and Related Natives in Novel Environments
Source: Front Microbiol. 2019 Mar 18;10:505. doi: 10.3389/fmicb.2019.00505 (PMC6431654; doi:10.3389/fmicb.2019.00505)
Supplement: Supplementary file 1 [file Data_Sheet_1.pdf]

Table S1. List of plant genera and species used in the experiment and the coordinates of the field locations where soils were sampled

Genera	Species	Origin	Site	Coordinates
Centaurea	*jacea*	Native	1	N51° 52.076' E5° 59.529'
			2	N51° 52.764' E6° 00.292'
			3	N51° 52.985' E5° 42.911'
			4	N51° 52.787' E5° 43.751'
			5	N51° 52.036' E6° 01.505'
	stoebe *	Range-expander	1	N51° 51.599' E5° 53.332'
			2	N51° 51.605' E5° 53.332'
			3	N51° 51.606' E5° 53.342'
			4	N51° 51.605' E5° 53.354'
			5	N51° 51.609' E5° 53.350'
Geranium	*molle*	Native	1	N51° 46.700' E5° 55.612'
			2	N51° 52.006' E5° 59.429'
			3	N51° 52.743' E6° 00.267'
			4	N51° 52.627' E6° 00.032'
			5	N51° 52.641' E6° 00.120'
	pyrenaicum	Range-expander	1	N51° 57.872' E5° 40.861'
			2	N51° 46.702' E5° 55.579'
			3	N51° 52.662' E6° 01.296'
			4	N51° 51.856' E6° 00.938'
			5	N51° 51.822' E6° 00.953'
Tragopogon	*pratensis*	Native	1	N51° 52.972' E5° 42.850'
			2	N51° 52.869' E5° 43.318'
			3	N51° 52.797' E5° 43.466'
			4	N51° 52.781' E5° 43.731'
			5	N51° 52.468' E5° 46.793'
	dubius *	Range-expander	1	N51° 50.161' E5° 51.224'
			2	N51° 50.152' E5° 51.228'
			3	N51° 50.285' E5° 51.113'
			4	N51° 50.248' E5° 51.075'
			5	N51° 50.253' E5° 51.072'
Rorippa	*sylvestris*	Native	1	N51° 52.275' E5° 54.398'
			2	N51° 52.104' E5° 56.275'
			3	N51° 52.145' E5° 59.389'
			4	N51° 52.652' E6° 00.010'
			5	N51° 52.761' E6° 00.255'
	austriaca	Range-expander	1	N51° 52.316' E5° 54.397'
			2	N51° 52.177' E5° 56.197'
			3	N51° 52.057' E5° 59.462'
			4	N51° 52.204' E5° 59.468'
			5	N51° 52.239' E5° 59.495'

(*) Range-expanders Centaurea stoebe and Tragopogon dubius are rare in the Netherlands and therefore, field soils of these species were collected from 5 individuals that were maximally 100 m apart

Table S2. PERMANOVA test on Bray-Curtis dissimilarity matrix of bacterial communities in the field inocula soils (9999 permutations)

Factor	R^2	Signif.
Soil pH	0.188	***
Soil (NO_3- NO_2)-N	0.059	**
Soil NH_4-N	0.061	**
Soil PO_4	0.029	*
Soil CN ratio	0.065	**
Plant species	0.216	**

Significance levels: * p<0.05; ** p<0.01; *** p<0.001

Table S3. Effects of plant genus and plant origin and their interaction on soil abiotic parameters and bacterial community characteristics of the field soils were analyzed using 2-way ANOVA

	Plant genus		Plant origin		Genus * Origin	
Soil parameters	F	Signif.	F	Signif.	F	Signif.
pH	33.461	***	2.495	ns	2.641	.
Nitrate ((NO_3^--NO_2^-)-N)	6.705	**	5.344	*	1.667	ns
Ammonia (NH_4^+-N)	19.521	***	4.705	*	4.932	**
P-Olsen	6.196	**	0.715	ns	1.654	ns
C: N ratio	18.768	***	4.048	.	8.203	***
Bacterial community	F	Signif.	F	Signif.	F	Signif.
OTU richness	12.817	***	0.811	ns	0.756	ns
Diversity (H’)	11.129	***	2.249	ns	1.089	ns
Evenness	3.574	*	0.981	ns	1.259	ns

Significance levels: ns p>0.1; . p<0.1;* p<0.05; ** p<0.01; *** p<0.001; **** p<0.0001

Table S4. PERMANOVA test on Bray-Curtis dissimilarity matrix of bacterial community composition and community functioning of all experimental soils (9999 permutations)

	**Bacterial community**		**Community functioning**	
Factors	R^2	Signif.	R^2	Signif.
Plant genera (G)	0,041	***	0,010	ns
Soil (S)	0,019	***	0,024	*
Time point (T)	0,025	***	0,004	ns
Plant origin (O)	0,012	***	0,001	ns
G x S	0,081	***	0,004	ns
G x T	0,020	ns	0,003	ns
S x T	0,005	ns	0,000	ns
G x O	0,036	***	0,001	ns
S x O	0,010	***	0,000	ns
T x O	0,005	ns	0,000	ns
G x S x T	0,016	ns	0,000	ns
G x S x O	0,034	***	0,000	ns
G x T x P	0,015	ns	0,000	ns
S x T X O	0,004	ns	0,000	ns
G x S x T x O	0,014	ns	0,000	ns

Significance levels: ns p > 0.05; * p<0.05; ** p<0.01; *** p<0.001; **** p<0.0001

Table S5. PERMANOVA tests on Bray-Curtis dissimilarity matrix (9999 permutations) of bacterial community composition and community functioning in the “novel” and “own” experimental soils

			Centaurea		*Geranium*		*Tragopogon*		*Rorippa*	
	Soil inocula	Factor	R^2	Signif.	R^2	Signif.	R^2	Signif.	R^2	Signif.
Bacterial community	Novel soil	Plant origin (P)	0,026	ns	0,038	ns	0,026	ns	0,028	ns
		Time (T)	0,092	ns	0,100	*	0,097	*	0,079	ns
		P * T	0,047	ns	0,056	ns	0,062	ns	0,050	ns
	Own soil	Plant origin (P)	0,201	***	0,080	**	0,260	***	0,093	**
		Time (T)	0,065	ns	0,062	ns	0,061	ns	0,080	ns
		P * T	0,039	ns	0,037	ns	0,043	ns	0,038	ns
Community functioning	Novel soil	Plant origin (P)	0,001	ns	0,001	ns	0,001	ns	0,000	ns
		Time (T)	0,008	ns	0,005	ns	0,011	ns	0,010	ns
		P * T	0,000	ns	0,000	ns	0,001	ns	0,000	ns
	Own soil	Plant origin (P)	0,008	ns	0,002	ns	0,001	ns	0,001	ns
		Time (T)	0,010	ns	0,003	ns	0,007	ns	0,011	ns
		P * T	0,003	ns	0,000	ns	-0,001	ns	0,002	ns

Significance levels: ns p > 0.05; * p<0.05; ** p<0.01; *** p<0.001; **** p<0.0001

Table S6. Bacterial community richness, diversity (H’) and evenness in the rhizosphere of the experimental soils. Values are means ± SE (N=5). Values in bold indicate $P < 0.05$

			4-weeks			8-weeks			12-weeks		
Soil	*Plant genus*	*Plant origin*	OTU richness	OTU diversity (H')	Evenness	OTU richness	OTU diversity (H')	Evenness	OTU richness	OTU diversity (H')	Evenness
Own soil	*Centaurea*	Native	2927 ± 264	6.529 ± 0.128	0.819 ± 0.013	2256 ± 237	6.469 ± 0.131	0.840 ± 0.015	2440 ± 206	6.585 ± 0.083	0.846 ± 0.006
		Range-expander	2381 ± 251	6.119 ± 0.416	0.788 ± 0.049	2960 ± 228	6.593 ± 0.195	0.826 ± 0.019	2958 ± 70	6.635 ± 0.078	0.830 ± 0.009
	Geranium	Native	2926 ± 105	6.256 ± 0.070	0.784 ± 0.011	3135 ± 151	6.596 ± 0.061	0.820 ± 0.006	2199 ± 218	6.364 ± 0.043	0.830 ± 0.008
		Range-expander	2290 ± 436	6.311 ± 0.231	0.824 ± 0.014	2263 ± 440	6.188 ± 0.222	0.810 ± 0.010	2750 ± 214	6.344 ± 0.131	0.802 ± 0.009
	Tragopogon	Native	2594 ± 330	6.417 ± 0.176	0.820 ± 0.015	2516 ± 145	6.578 ± 0.073	0.841 ± 0.012	3024 ± 194	6.629 ± 0.050	0.828 ± 0.008
		Range-expander	2635 ± 239	6.317 ± 0.166	0.803 ± 0.015	1680 ± 309	5.952 ± 0.129	0.810 ± 0.011	2124 ± 129	5.977 ± 0.070	0.781 ± 0.010
	Rorippa	Native	1323 ± 95	5.380 ± 0.147	0.750 ± 0.023	1351 ± 216	5.264 ± 0.215	0.736 ± 0.027	1957 ± 334	5.593 ± 0.157	0.744 ± 0.005
		Range-expander	2152 ± 255	6.017 ± 0.266	0.785 ± 0.023	1915 ± 238	5.792 ± 0.297	0.768 ± 0.028	1826 ± 241	6.096 ± 0.215	0.815 ± 0.020
Novel soil	*Centaurea*	Native	2100 ± 265	6.202 ± 0.241	0.814 ± 0.027	2747 ± 253	6.307 ± 0.164	0.798 ± 0.012	2947 ± 459	6.406 ± 0.088	0.807 ± 0.010
		Range-expander	2960 ± 228	6.490 ± 0.333	0.812 ± 0.036	2483 ± 226	6.287 ± 0.187	0.807 ± 0.029	2621 ± 155	6.321 ± 0.175	0.804 ± 0.021
	Geranium	Native	2512 ± 400	6.471 ± 0.227	0.831 ± 0.018	2872 ± 525	6.415 ± 0.102	0.814 ± 0.014	3051 ± 470	6.272 ± 0.230	0.786 ± 0.020
		Range-expander	2796 ± 340	6.607 ± 0.142	0.835 ± 0.010	3068 ± 333	6.527 ± 0.101	0.816 ± 0.009	3307 ± 390	6.475 ± 0.210	0.801 ± 0.019
	Tragopogon	Native	2714 ± 180	6.443 ± 0.082	0.816 ± 0.012	3640 ± 466	6.608 ± 0.098	0.810 ± 0.017	2614 ± 256	6.283 ± 0.276	0.799 ± 0.026
		Range-expander	3452 ± 320	6.648 ± 0.068	0.818 ± 0.009	2952 ± 301	6.523 ± 0.100	0.819 ± 0.012	2578 ± 245	6.430 ± 0.150	0.820 ± 0.013
	Rorippa	Native	2680 ± 203	6.400 ± 0.139	0.812 ± 0.017	2992 ± 211	6.447 ± 0.152	0.806 ± 0.017	3289 ± 368	6.592 ± 0.152	0.816 ± 0.009
		Range-expander	2985 ± 195	6.492 ± 0.134	0.812 ± 0.016	3966 ± 751	6.755 ± 0.075	0.822 ± 0.017	3290 ± 177	6.695 ± 0.116	0.827 ± 0.010
Fixed factors	Plant genera	*F value*	3.643	2.733	1.651	1.154	2.241	3.558	1.256	1.062	2.018
		p-value	**0.017**	0.051	0.187	0.334	0.092	**0.019**	0.297	0.372	0.121
	Plant origin	*F value*	3.530	2.700	0.337	0.136	0.004	0.017	0.049	0.102	0.127
		p-value	0.065	0.105	0.563	0.713	0.949	0.894	0.824	0.750	0.722
	Soil	*F value*	9.271	10.010	4.893	26.385	10.717	0.108	16.687	4.451	0.168
		p-value	**0.003**	**0.002**	**0.030**	**<0.001**	**0.001**	0.743	**<0.001**	**0.039**	0.683
	Genera*origin	*F value*	2.166	0.256	0.630	4.395	4.604	0.871	1.742	1.996	3.671
		p-value	0.101	0.856	0.598	**0.007**	**0.005**	0.461	0.168	0.124	**0.017**
	Genera*soil	*F value*	5.361	1.803	0.876	6.922	10.952	5.023	7.566	9.002	5.931
		p-value	**0.002**	0.156	0.458	**<0.001**	**<0.001**	**0.003**	**<0.001**	**<0.001**	**0.001**
	Origin*soil	*F value*	4.117	1.023	0.217	0.283	2.173	1.343	0.000	1.145	1.960
		p-value	**0.047**	0.315	0.642	0.596	0.145	0.251	0.977	0.288	0.166
	Gen*ori*soil	*F value*	3.684	1.521	0.639	2.291	1.550	0.509	1.916	2.397	4.016
		p-value	**0.016**	0.218	0.592	0.086	0.211	0.677	0.137	0.077	**0.011**

SI Figure captions

Figure S1. Principal Coordinate Analyses of the bacterial community of field soils, which originate from locations where the plant species grown in the field and served as inocula in our experiment. Soil abiotic properties are projected in the ordination plot as supplementary variables (arrows). Symbols are means ± SE (N=5). Different colours represent the different plant pairs of range expander and congeneric natives. Within each pair, circles represent the native plant species and triangles the range-expander.

Figure S2. Soil properties of the soils collected from field populations, and used as inocula in the experiment. White bars represent native plant species and grey bars represent range-expanding plant species in each plant genus. (**A**) pH, (**B**) nitrate and nitrite availability, (**C**) ammonium availability, (**D**) phosphate availability, (**E**) soil C: N ratio, (**F**) 16S OTU richness, (**G**) OTU Shannon diversity index, H' [(1)] and (**H**) community evenness, E_H [(2)]. Bars are means ± SE (N=5). Asterisk (*) and dot (.) symbols indicate $P < 0.05$ and $P < 0.1$, respectively, in pairwise within-genus comparisons. [(1)] Shannon diversity index $H' = -\sum_{i=1}^{n} p_i ln\, p_i$ where pi is the proportion of species *i* relative to the total number of species. [(2)] Community evenness $E_H = H/\ln S$ where S is the total number of OTUs.

Figure S3. Principal Coordinate Analyses of the rhizosphere bacterial community composition for each pair of a range-expander and its congeneric native plant species (A: *Centaurea*, B: *Geranium*, C: *Tragopogon*, D: *Rorippa*) grown in "novel" soils. The symbols are means ± SE (N=5). Within each pair, circles represent the native plant species and triangles the range-expander. Colors indicate time of harvest (4, 8 and 12 weeks) as noted in the legend.

Figure S4. Principal Coordinate Analyses of the rhizosphere bacterial community composition for each pair of a range-expander and its congeneric native plant species (A: *Centaurea*, B: *Geranium*, C: *Tragopogon*, D: *Rorippa*) grown in "own" soils. The symbols are means ± SE (N=5). Within each pair, circles represent the native plant species and triangles the range-expander. Colors indicate time of harvest (4, 8 and 12 weeks) as noted in the legend.

Figure S5. Principal Coordinate Analyses of the catabolic response profiles for each pair of a range-expander and its congeneric native plant species (A: *Centaurea*, B: *Geranium*, C: *Tragopogon*, D: *Rorippa*) grown in "novel" soils. The symbols are means ± SE (N=5). Within each pair, circles represent the native plant species and triangles the range-expander. Arrows representing each substrate are displayed over the ordination plot as supplementary variables. Colors indicate time of harvest (4, 8 and 12 weeks) as noted in the legend.

Figure S6. Principal Coordinate Analyses of the catabolic response profiles for each pair of a range-expander and its congeneric native plant species (A: *Centaurea*, B: *Geranium*, C: *Tragopogon*, D: *Rorippa*) grown in "own" soils. The symbols are means ± SE (N=5). Within each pair, circles represent the native plant species and triangles the range-expander. Arrows representing each substrate are displayed over the ordination plot as supplementary variables. Colors indicate time of harvest (4, 8 and 12 weeks) as noted in the legend.

Figure S1

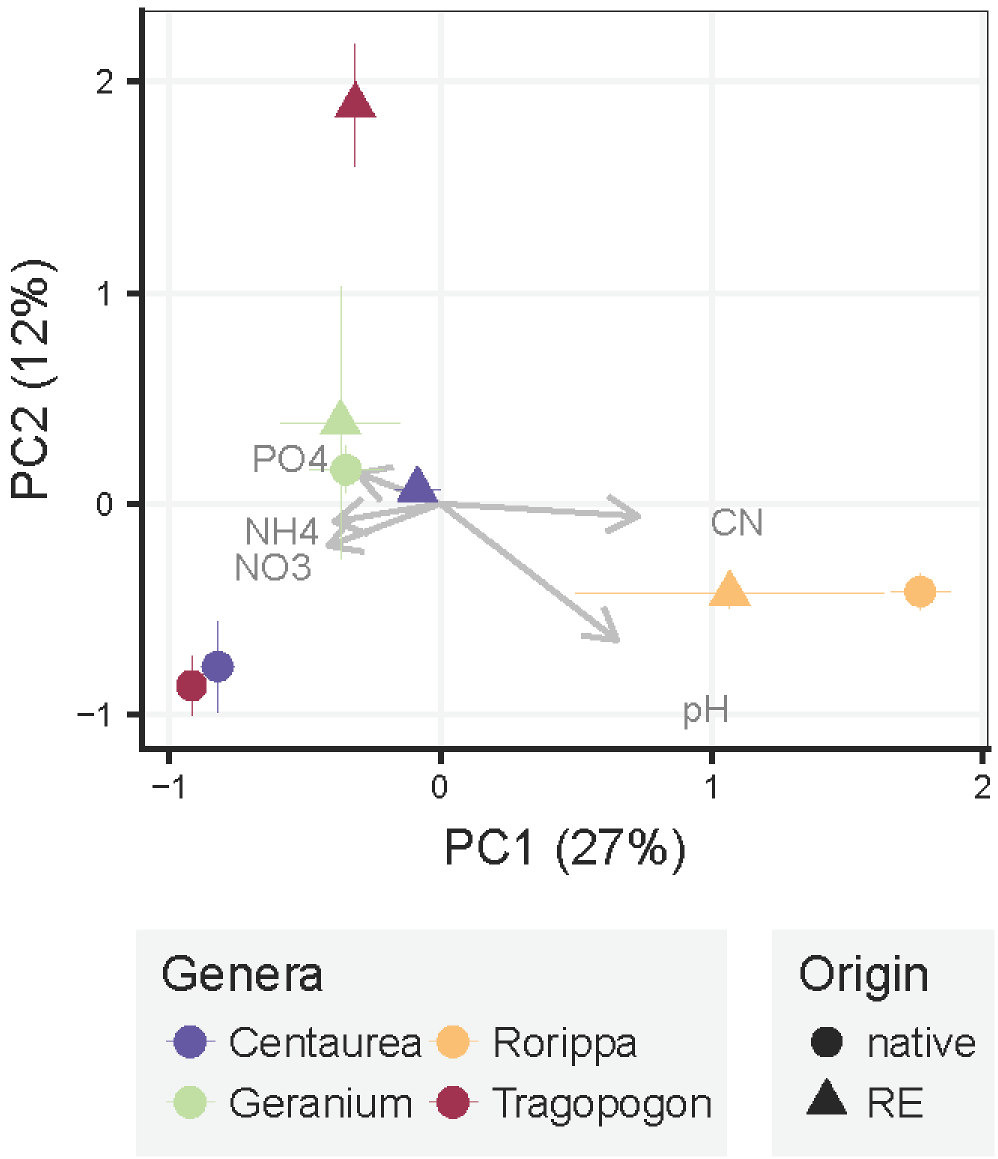

PC2 (12%)
2
1
0
−1
−1
0
1
2
PC1 (27%)
PO4
NH4
NO3
CN
pH
Genera
Centaurea
Rorippa
Geranium
Tragopogon
Origin
native
RE

Figure S2

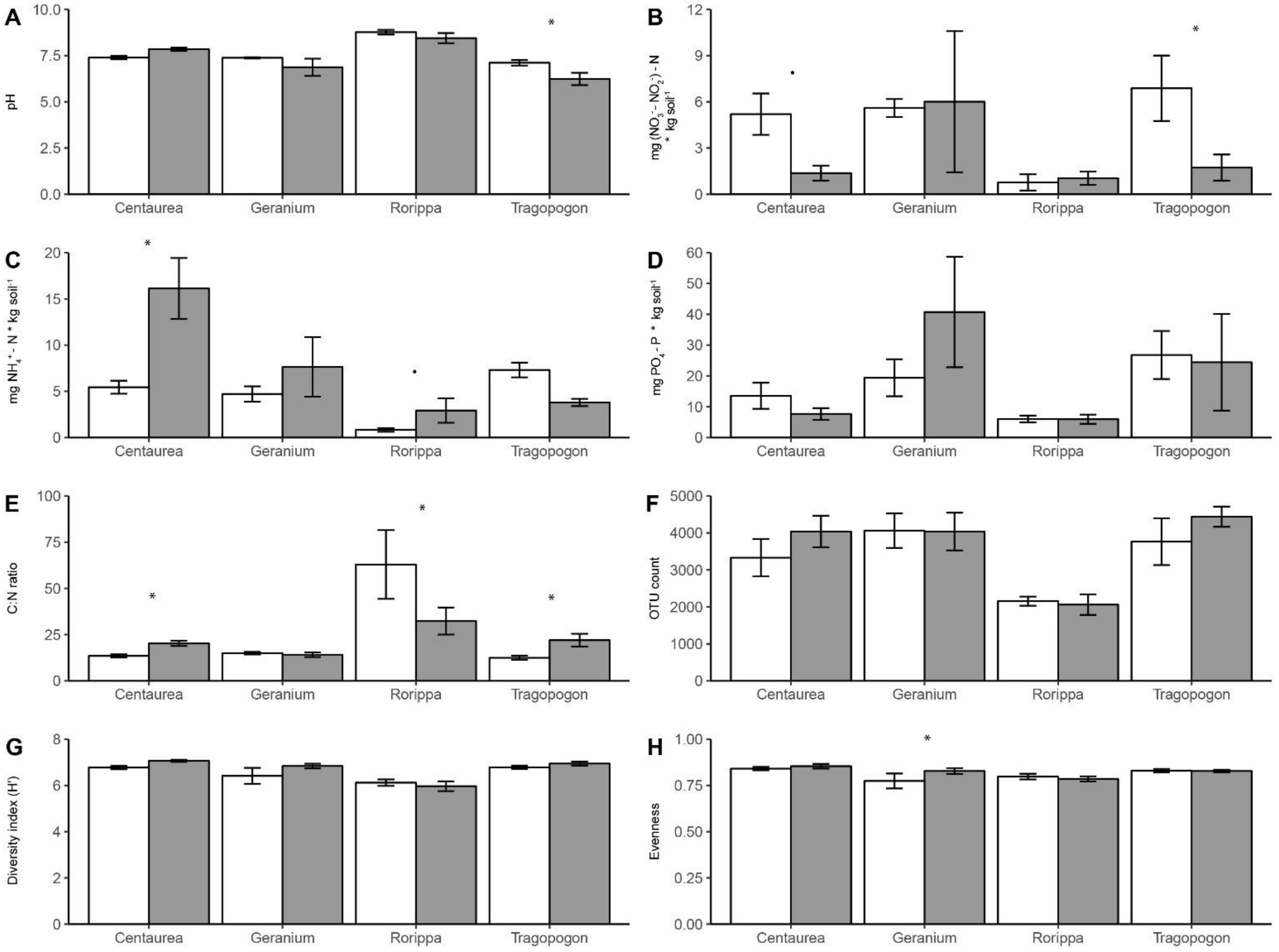

A
10.0
7.5
5.0
2.5
0.0
pH
*
Centaurea
Geranium
Rorippa
Tragopogon
B
12
9
6
3
0
mg (NO3- - NO2-) - N * kg soil-1
*
C
20
15
10
5
0
mg NH4+ - N * kg soil-1
*
D
60
50
40
30
20
10
0
mg PO4 - P * kg soil-1
E
100
75
50
25
0
C:N ratio
*
*
*
F
5000
4000
3000
2000
1000
0
OTU count
G
8
6
4
2
0
Diversity index (H')
H
1.00
0.75
0.50
0.25
0.00
Evenness
*

Figure S3

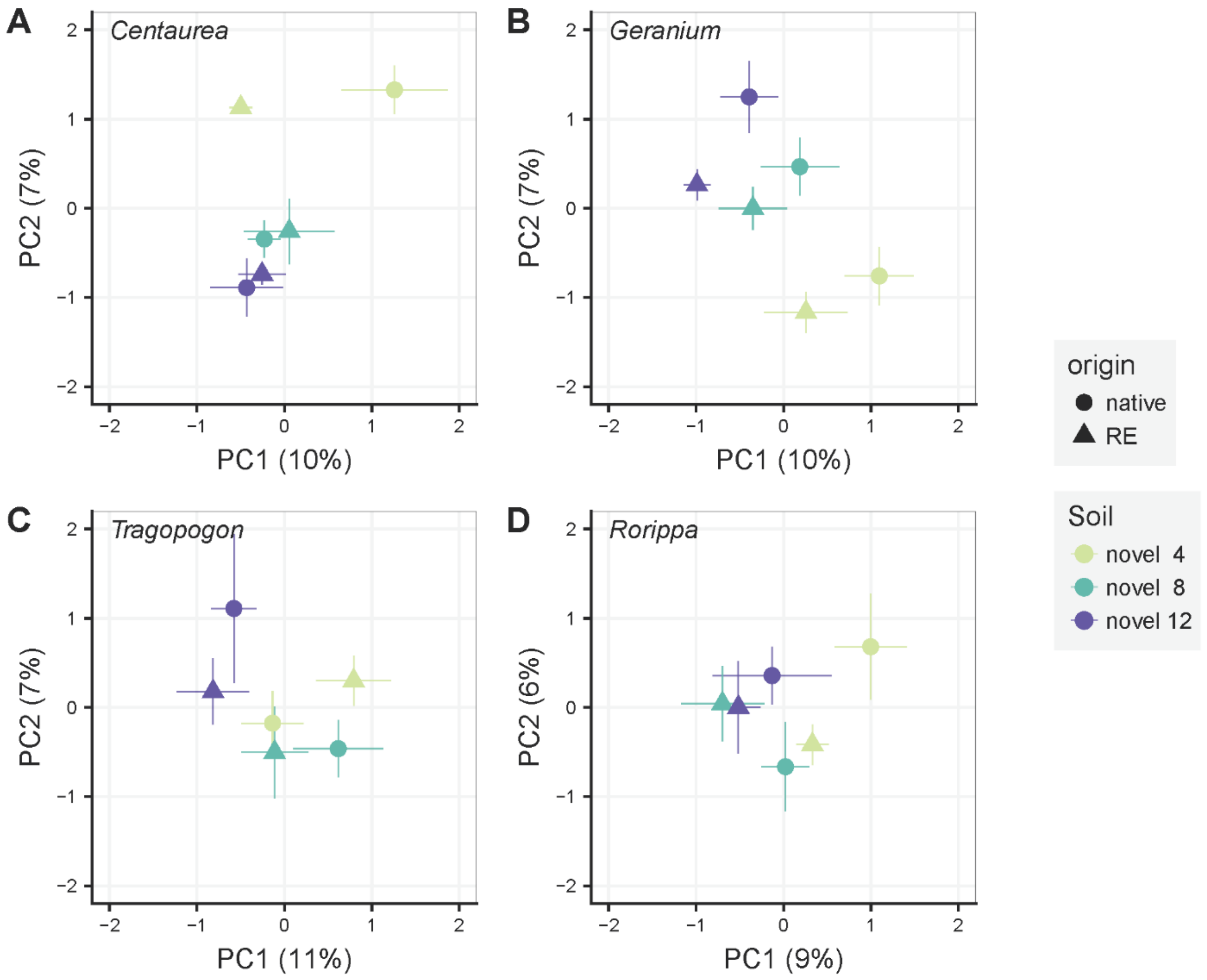
A
Centaurea
PC2 (7%)
PC1 (10%)
B
Geranium
PC2 (7%)
PC1 (10%)
C
Tragopogon
PC2 (7%)
PC1 (11%)
D
Rorippa
PC2 (6%)
PC1 (9%)
origin
native
RE
Soil
novel 4
novel 8
novel 12

Figure S4

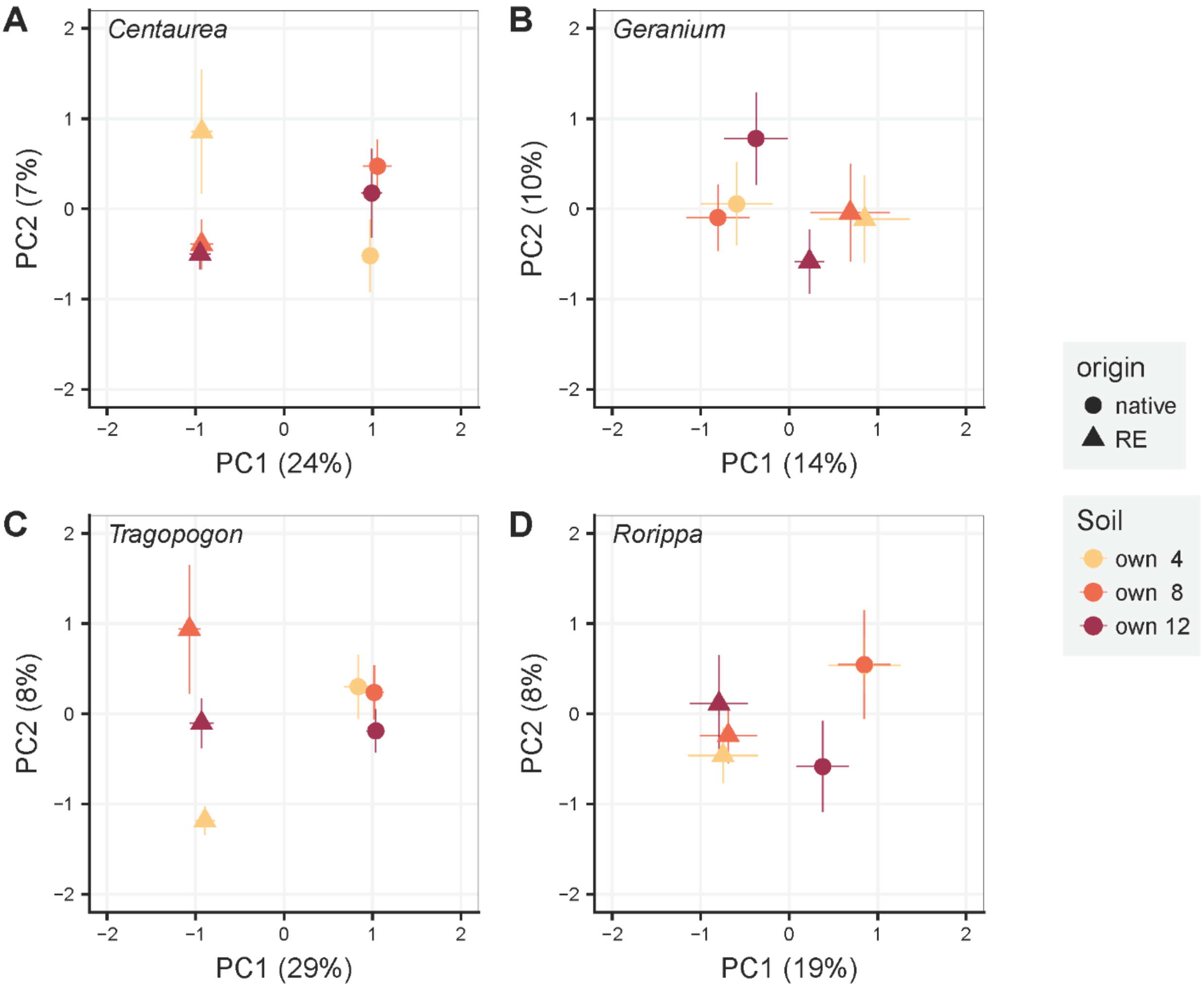
A
Centaurea
PC2 (7%)
PC1 (24%)
B
Geranium
PC2 (10%)
PC1 (14%)
C
Tragopogon
PC2 (8%)
PC1 (29%)
D
Rorippa
PC2 (8%)
PC1 (19%)
origin
native
RE
Soil
own 4
own 8
own 12

Figure S5

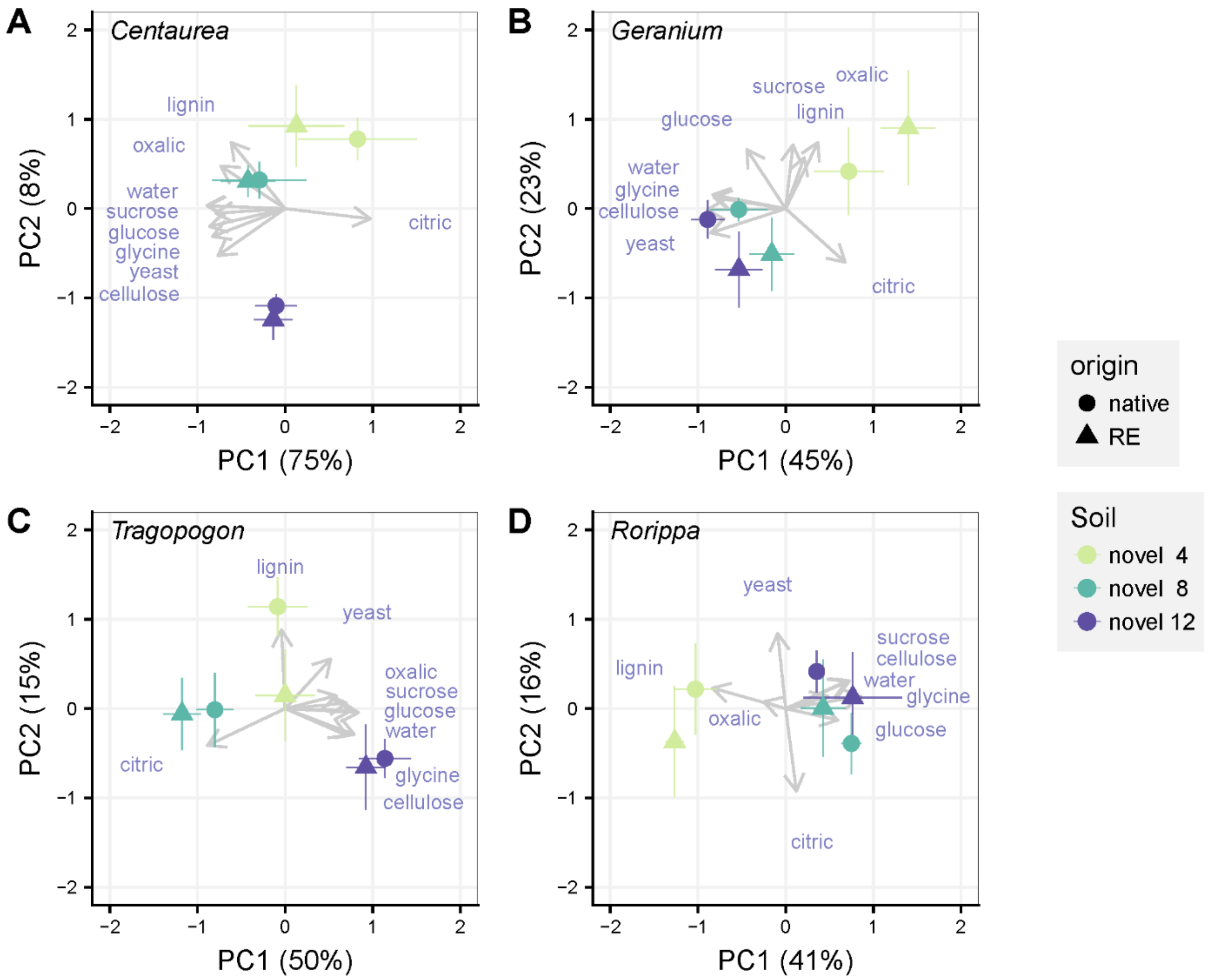
A
Centaurea
lignin
oxalic
water
sucrose
glucose
glycine
yeast
cellulose
citric
PC2 (8%)
PC1 (75%)
B
Geranium
sucrose
oxalic
lignin
glucose
water
glycine
cellulose
yeast
citric
PC2 (23%)
PC1 (45%)
C
Tragopogon
lignin
yeast
oxalic
sucrose
glucose
water
glycine
cellulose
citric
PC2 (15%)
PC1 (50%)
D
Rorippa
yeast
lignin
sucrose
cellulose
water
glycine
glucose
oxalic
citric
PC2 (16%)
PC1 (41%)
origin
native
RE
Soil
novel 4
novel 8
novel 12

Figure S6

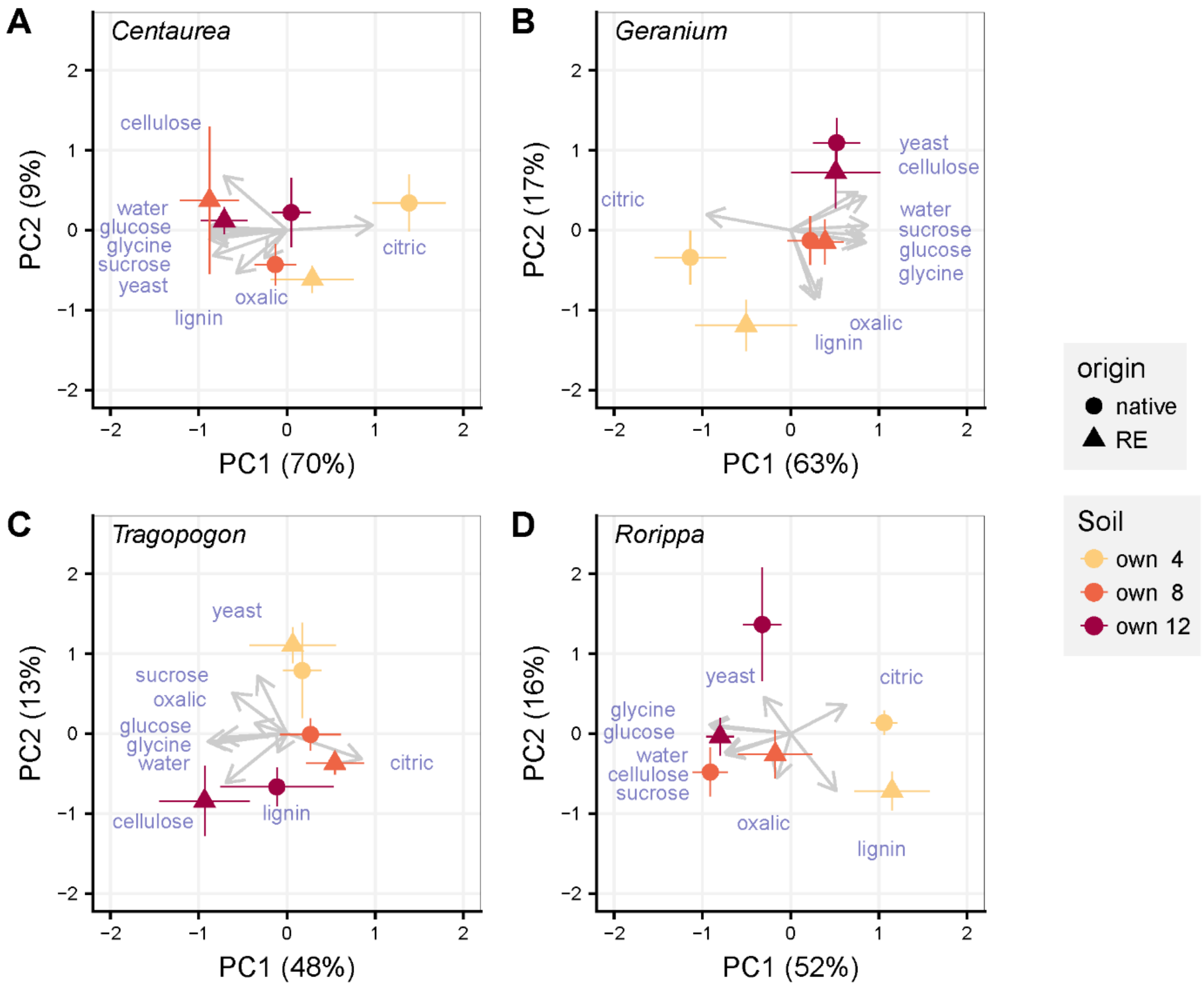
A
Centaurea
PC2 (9%)
PC1 (70%)
cellulose
water
glucose
glycine
sucrose
yeast
lignin
oxalic
citric
B
Geranium
PC2 (17%)
PC1 (63%)
yeast
cellulose
citric
water
sucrose
glucose
glycine
oxalic
lignin
C
Tragopogon
PC2 (13%)
PC1 (48%)
yeast
sucrose
oxalic
glucose
glycine
water
citric
cellulose
lignin
D
Rorippa
PC2 (16%)
PC1 (52%)
yeast
citric
glycine
glucose
water
cellulose
sucrose
oxalic
lignin
origin
native
RE
Soil
own 4
own 8
own 12
